# Supplementary material for: Human Rotaviruses of Multiple Genotypes Acquire Conserved VP4 Mutations during Serial Passage
Source: Viruses. 2024 Jun 18;16(6):978. doi: 10.3390/v16060978 (PMC11209247; doi:10.3390/v16060978)
Supplement: Supplementary file 1 [file viruses-16-00978-s001.zip › Figure S1.Sequence coverage.pdf]

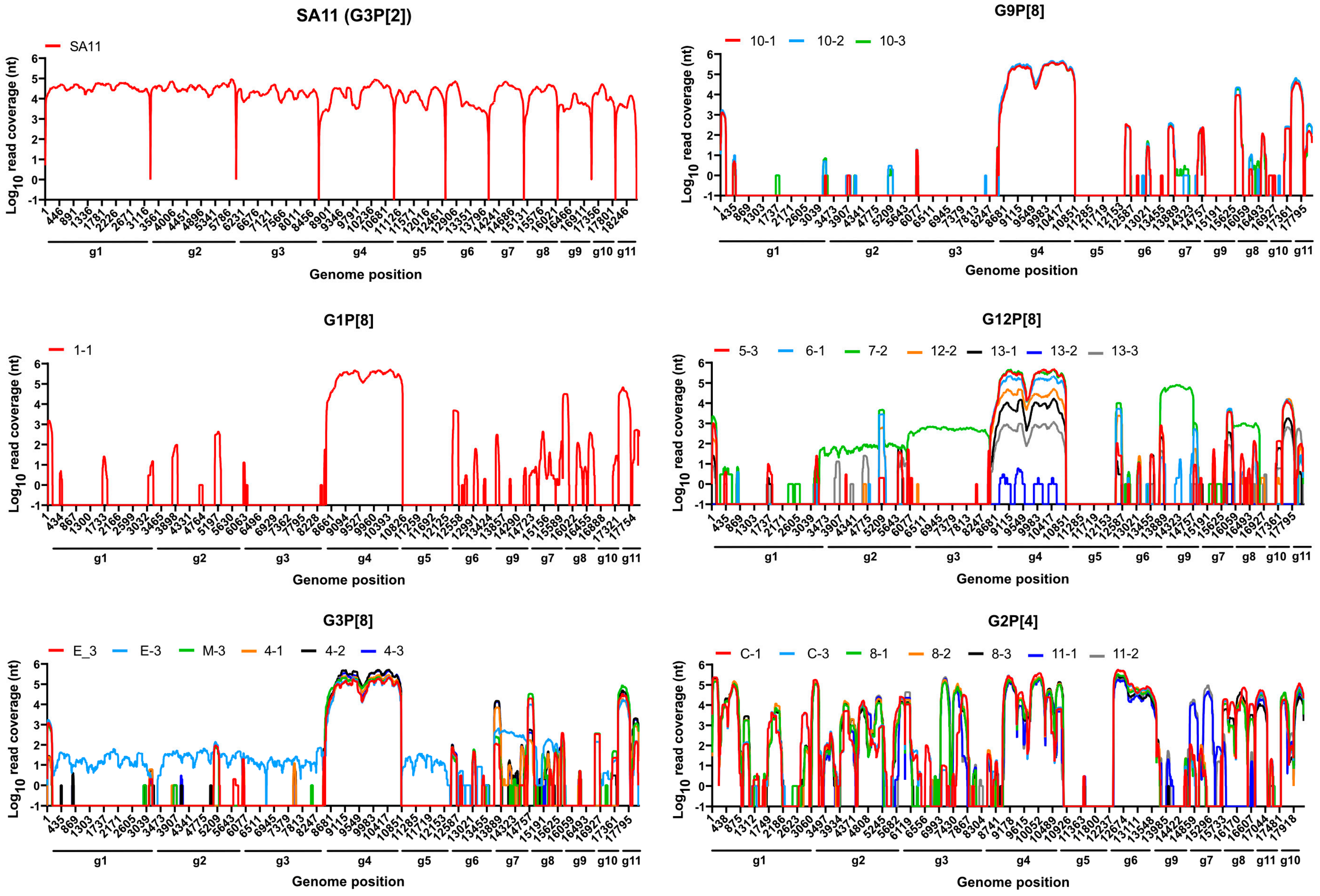

Figure S1. Nucleotide coverage by genotype. Each graph represents a distinct rotavirus genotype. The *x*-axis indicates nucleotide position in a concatenated rotavirus genome, with individual genome segments indicated below. The *y*-axis indicates Illumina read counts. Different line colors indicate distinct specimens or lineages, whose names are shown in the format of the "short names" listed in Table 1. In most cases, coverage is highest for g4, which encodes cell attachment protein VP4.
